# Gradual increases in light intensity and photoperiod enhance light use efficiency and dry matter in indoor basil

**DOI:** 10.3389/fpls.2026.1861031

**Published:** 2026-06-12

**Authors:** Nazmin Akter, Md Shamim Ahamed, Gail Taylor, Laura Cammarisano

**Affiliations:** 1 Department of Plant Sciences, University of California, Davis, Davis, CA, United States; 2 Department of Biological and Agricultural Engineering, University of California, Davis, Davis, CA, United States; 3 Department of Botany, University College of London, London, United Kingdom

**Keywords:** dry biomass, dynamic lighting, indoor vertical farming, light use efficiency, morphology, stomatal conductance

## Abstract

Basil (*Ocimum basilicum* L. Genovese) is a highly valued and economically important herb with significant culinary qualities. The quantity of light supplied to plants, including both light intensity and photoperiod, plays a critical role in regulating plant morphology and biomass accumulation. Optimizing these factors can simultaneously enhance yield and resource efficiency. This study assessed the effects of constant versus gradually increasing light intensity and photoperiod on basil growth, physiology, and light use efficiency (LUE), while maintaining an equivalent average daily light integral (DLI) over the 24-day growing period. Four different treatments were applied in a climate-controlled growth chamber: (CIP) constant light intensity (300 μmol m^-^² s^-^¹) and constant photoperiod (16 h), (CIDP) constant light intensity with a dynamic photoperiod (14-16–18 h), (DICP) dynamic light intensity (200-300-400 μmol m^-^² s^-^¹) with constant photoperiod, and (DIP) dynamic light intensity (200-300-400 μmol m^-^² s^-^¹) and photoperiod (14-16–18 h) over time. In comparison to CIP, treatment DIP resulted in a 9% increase in both dry weight and LUE, and a 19% increase in non-destructive chlorophyll content, whereas stomatal conductance was 25% higher in CIP. CIDP exhibited the lowest values for leaf area, fresh weight, dry weight, LUE, destructive chlorophyll and carotenoid content, and non-photochemical quenching (NPQ). These results suggest that dynamic light strategies can improve LUE and dry matter accumulation under comparable average DLI conditions. Future research should investigate whether these responses are associated with any changes in postharvest quality, processing characteristics, and the temporal dynamics of secondary metabolites to further refine lighting strategies for indoor farming.

## 1 Introduction

The strategic control of light in controlled environment agriculture (CEA) systems, such as indoor vertical farms and greenhouses, is key to optimizing plant productivity and resource use efficiency. Light is the source of energy for photosynthesis as well as an effective environmental cue that regulates plant morphology, physiology, and secondary metabolism (Dou et al., 2019; Lin et al., 2021; Ryu et al., 2024). In indoor farming systems, artificial lighting is the only source of illumination, and light intensity, spectral quality, and photoperiod play a critical role influencing plant growth and development (Avgoustaki, 2019; Solouki et al., 2023). Light intensity is the amount of light received per unit area and is typically measured as photon flux density (PFD, in µmol m^-^² s^-^¹). It directly affects the photosynthetic rate and biomass yield. Light intensity and photoperiod are two key factors that can increase the total amount of light delivered to plants each day. Higher PFDs are beneficial for photosynthetic efficiency, leaf thickness, and dry matter accumulation (Dou et al., 2019; Sutulienė et al., 2022). However, excessive light exposure above the leaf-level photosynthetic light saturation point can lead not only to photoinhibition and oxidative stress but also to reduced whole-canopy light use efficiency, as surplus absorbed light cannot be efficiently utilized for carbon assimilation under continuous high PFD conditions (Beaman et al., 2009). The additional light also increases the thermal loads for cooling the controlled cultivation space, as well as increasing evapotranspiration rates. Additionally, the light saturation point varies by species and cultivar, necessitating careful management in CEA systems. On the other hand, low light levels can limit growth by creating energy deficits, leading to weak plants that produce lower yields and lower product quality (Aldarkazali, 2020; Tabbert et al., 2022). Photoperiod (the duration of light exposure within a 24-h day) is another important factor affecting plant growth and development. Through photoreceptors that detect daylength and initiate hormonal signaling pathways, photoperiod controls processes such as flowering, stem elongation, biomass partitioning, and circadian rhythms (Avgoustaki, 2019; Eghbal et al., 2024). The optimal combination of light intensity and photoperiod to a target daily light integral (DLI) is critical for crop performance and energy efficiency.

Basil (*Ocimum basilicum* L. Genovese), a popularly grown aromatic herb, is valued for its culinary, therapeutic, and decorative uses (Nadeem et al., 2019). Due to its short growth cycle, compact canopy architecture, and high sensitivity to environmental conditions, it is particularly well-suited for CEA cultivation (Chang et al., 2008; Dou et al., 2019). Light intensity and photoperiod have been shown to have a significant impact on basil’s growth rate, biomass accumulation, and secondary metabolism, which includes the biosynthesis of essential oils, phenolic compounds, antioxidants, and volatiles that give it its distinctive flavor and therapeutic value (Avgoustaki, 2019; Eghbal et al., 2024; Larsen et al., 2020). For basil, extended photoperiods are known to enhance vegetative growth, delay flowering, and improve leaf area and biomass accumulation, which are desirable traits in the market (Dou et al., 2019; Walters and Currey, 2018). However, excessively long photoperiods, particularly when intense, i.e., combined with high PFDs, can lead to imbalanced metabolic activity, increased transpiration, and photodamage (van Iersel and Gianino, 2017).

In indoor cultivation, fixed light regimes involving constant intensity and photoperiod are commonly used. While straightforward to implement, such constant lighting schemes do not replicate the dynamic fluctuations found in natural environments, may not align with plant light requirements, and can result in suboptimal plant performance and inefficient energy use. Persistent high light can induce photoinhibition, elevate leaf temperature, and result in excessive energy consumption without proportional yield gains (Horton, 2012; Van Brenk et al., 2025). Furthermore, static light regimes may suppress stress-induced metabolic pathways essential for the production of bioactive compounds such as flavonoids (Solouki et al., 2023). In contrast, dynamic lighting (featuring temporal changes in light intensity and/or photoperiod) has gained attention as a promising strategy to improve light use efficiency (LUE) and support normal plant development. Dynamic lighting strategies may include short-term fluctuations in light conditions within a day, as well as adjustments based on the plant developmental stage, in which light intensity and/or photoperiod are gradually modified across crop growth stages. The present study focuses on developmental stage-based lighting adjustments designed to better match changing plant light demand during canopy development. Such regimes simulate light fluctuations and allow for recovery periods, thereby minimizing stress and optimizing resource utilization (Park and Runkle, 2018; van Delden et al., 2021). Temporal modulation of light intensity has been shown to enhance biomass accumulation, photosynthetic rates, and stress tolerance in various crops, including tomato, lettuce, basil, and cucumber (Aliniaeifard, 2024; Hammock and Sams, 2023). However, plant responses to dynamic lighting are highly dependent on genotype, developmental stage, and light history, suggesting a need for crop- and stage-specific “light recipes” (Bantis et al., 2016; Dou et al., 2019). Advances in LED technology have enabled the design of programmable lighting systems capable of delivering finely tuned, dynamic light profiles without major infrastructure modifications, opening new frontiers in sustainable CEA (Arcel et al., 2021; Paradiso and Proietti, 2022).

Despite increasing interest in adaptive lighting strategies, limited research has examined the combined effect of temporally dynamic light intensity and photoperiod on basil when the total DLI remains constant at the end of cultivation. Previous studies have focused on static conditions or single effects of either light intensity or photoperiod. A study by Jin et al. (2023) demonstrated that gradually increasing light intensity during lettuce cultivation led to 13-16% greater shoot dry weight and improved LUE compared to constant or decreasing light regimes, despite all treatments receiving the same total daily light integral. The improved performance was attributed to greater light interception during later growth stages when canopy coverage was highest, highlighting the potential benefits of dynamic lighting strategies in CEA. Another study by Eghbal et al. (2024) showed that varying light intensity with far-red spectrum and photoperiod, under a constant DLI, significantly influenced basil growth, physiology, and metabolite production. Longer photoperiods at lower intensity increased biomass and chlorophyll content, whereas shorter photoperiods at higher intensity increased antioxidant activity. Similarly, Hammock and Sams (2023) showed that timing and duration of supplemental LED and HPS lighting influenced basil yield and LUE across seasons, yet such studies have not tested coordinated temporal changes in both intensity and photoperiod under controlled conditions with constant DLI.

This study aimed to evaluate the effects of constant versus dynamic (gradually increasing) light intensity and photoperiod on the morphological and physiological responses of basil, focusing on LUE, dry matter accumulation, and photo-physiological responses. We hypothesized that synchronizing gradual increases in light intensity and photoperiod over time, while maintaining a consistent DLI, would enhance LUE and support favorable physiological acclimation in basil, compared with constant light. To test this hypothesis, basil plants were exposed to temporal increases in PFD and photoperiod, separately and simultaneously, while maintaining comparable cumulative DLI.

## 2 Materials and methods

### 2.1 Pre-transplant growth conditions

**Figure 1:**
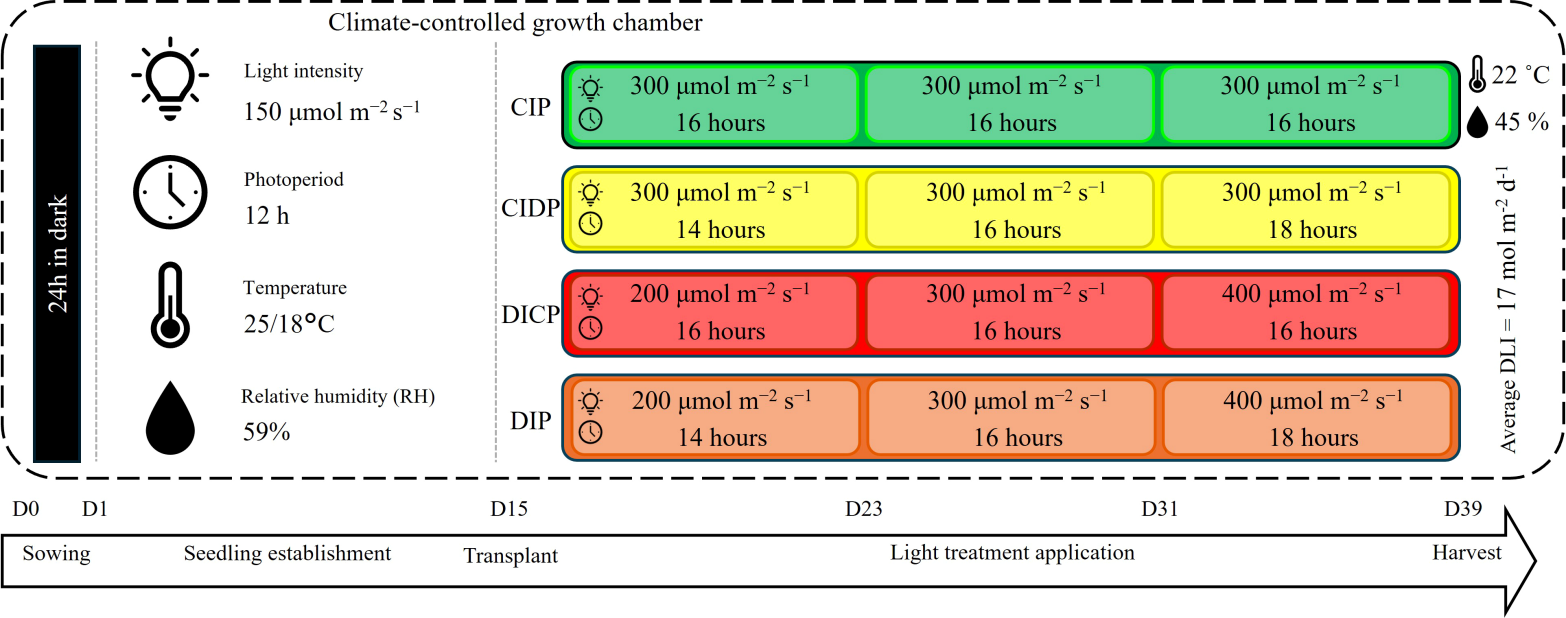
Experimental design illustrating environmental conditions in a climate-controlled growth chamber from sowing (day 0, D0) through transplant (D15) to harvest (D39). Four light regimes (CIP, green; CIDP, yellow; DICP, red; DIP, orange), combining constant or gradually increasing light intensity and photoperiod while maintaining equal daily light integral (DLI), were applied from D15 to D39 to basil plantlets cv. ‘Genovese’.

This study was conducted in a climate-controlled growth chamber at the Department of Plant Sciences, UC Davis, from February 23 to April 2, 2025 (first replication), and from March 26 to May 3, 2025 (second replication). Basil (*Ocimum basilicum* L. cv. ‘Genovese’) seeds (Johnny’s Selected Seeds, Winslow, Maine, USA) were sown in OrganiPlug cubes. Plugs were 1.25 inches × 1.25 inches (Ventana Plant Science, USA) and were made from a mixture of coco coir, peat moss, biochar and Trichoderma (ingredients approved by the Organic Materials Review Institute (OMRI) for use in certified organic production). Each seed was placed in an individual plug and kept in a dark environment at room temperature for 24 hours. Afterward, the plugs were exposed to light conditions in the growth chamber for two weeks before transferring to the experimental setup (**Figure 1**). The light intensity was maintained at 150 µmol m^-^² s^-^¹ with a 12-hour photoperiod. The average temperature of the chamber was maintained at 25 ± 0.5 °C, and the relative humidity at 59 ± 1%, monitored using HOBO MX1101 dataloggers (Onset Computer Corporation, MA, USA). CO_2_ concentration was neither controlled nor measured. All treatments were conducted within the same climate-controlled chamber under comparable environmental conditions, and measurement plants were selected from the most spatially uniform areas identified through light mapping (**Supplementary Figure 1**) to minimize positional variability. In addition, treatment locations within the chamber were rotated between temporal replications to minimize potential positional effects associated with airflow, temperature, and CO_2_. Hoagland’s nutrient solution, with a controlled electrical conductivity (EC) of 1.8 mS/cm and a pH of 6.5, was used for irrigation. Seedlings were irrigated twice a day manually during this establishment phase.

### 2.2 Description of light treatments

Uniform seedlings with two true leaves (15 days after sowing) were placed in the treatment setup. Each plug was placed in larger rockwool cubes (4 cm × 4 cm × 3 cm, L × W × H), and a drip irrigation system was used to irrigate. The cubes were located on a large table divided into four areas, separated by white reflective plastic. Each block hosted a different light treatment and 18 equally spaced plants. The planting density in this study was approximately 21 plants per square meter. Four light treatments, including one constant and three dynamic treatments, were evaluated as detailed in **Table 1**. The constant intensity and photoperiod treatment (CIP) was characterized by a fixed PFD of 300 µmol m^-^² s^-^¹ and a constant 16 h photoperiod. The three dynamic lighting treatments included constant intensity with dynamic photoperiod (CIDP; 300 µmol m^-^² s^-^¹ with photoperiods of 14, 16, and 18 h), dynamic intensity with constant photoperiod (DICP; PFDs of 200, 300, and 400 µmol m^-^² s^-^¹ with a constant 16 h photoperiod), and dynamic intensity and photoperiod (DIP; PFDs of 200, 300, and 400 µmol m^-^² s^-^¹ combined with photoperiods of 14, 16, and 18 h). The DLI was the same across all treatments (17 mol m^-2^ d^-1^) at the end of the cultivation period, and changes in the dynamic treatments were implemented every 8-day intervals. The whole treatment period of this study was 24 days, whereas the total growing cycle was 39 days from sowing (**Figure 1**). The selected PFD range (200-400 μmol m^-^² s^-^¹) and photoperiod range (14–18 h) were chosen to represent moderate-to-high lighting conditions commonly used for indoor basil production while maintaining a comparable average DLI across treatments (Dou et al., 2018). The gradual 8-day shifts were designed to reflect increasing canopy development and plant light demand during basil growth and to evaluate stage-dependent responses to dynamic lighting (Jin et al., 2023). The light was provided by full-spectrum (spectral composition: 13% between 400 and 499 nm, 37% between 500 and 599 nm, 44% between 600 and 699 nm, 6% between 700 and 780 nm) LED lamps (SPYD 2pi, Fluence Bioengineering Inc., Texas, USA) positioned 132 cm above the cultivation floor. Measurement plants were positioned in the most uniform area of the trays, identified by mapping PFD at 18 different plant positions using a spectroradiometer (PG200N, UPRtek, Taiwan) just above the rockwool surface. Measurement locations are shown as a heatmap (**Supplementary Figure 1**) using the average light intensity for each treatment. **Table 2** presents the light measurements and corresponding photoperiod data for each treatment at 8-day intervals.

**Table 1:** Target photon flux density (PFD), photoperiod, and average daily light integral (DLI) for the four light treatments (CIP, CIDP, DICP, and DIP).

Treatments	PFD (μmol m^-^² s^-^¹)	Photoperiod (hours)	Average DLI (mol m^-^² day^-^¹)
CIP (Constant Intensity and Photoperiod)	300	16	17
CIDP (Constant Intensity and Dynamic Photoperiod)	300	14-16-18	17
DICP (Dynamic Intensity and Constant Photoperiod)	200-300-400	16	17
DIP (Dynamic Intensity and Photoperiod)	200-300-400	14-16-18	17

Treatments were designed to achieve a comparable average DLI over the cultivation period.

**Table 2:** Measured photon flux density (PFD) and photoperiod across three 8-day intervals for the four light treatments (CIP, Constant Intensity and Photoperiod, CIDP, Constant Intensity and Dynamic Photoperiod, DICP, Dynamic Intensity and Constant Photoperiod, DIP, Dynamic Intensity and Photoperiod).

Treatments	PFD 1^st^ 8 days (μmol m⁻² s⁻¹)	PFD 2^nd^ 8 days (μmol m⁻² s⁻¹)	PFD 3^rd^ 8 days (μmol m⁻² s⁻¹)	PFD 1^st^ 8 days (hours)	PFD 2^nd^ 8 days (hours)	PFD 3^rd^ 8 days (hours)
CIP	274 ± 11	274 ± 11	274 ± 11	16	16	16
CIDP	278 ± 14	278 ± 14	278 ± 14	14	16	18
DICP	181 ± 8	281 ± 10	379 ± 20	16	16	16
DIP	191 ± 12	272 ± 22	398 ± 29	14	16	18

Data are reported as mean ± SE and represent the average of weekly measurements taken at the substrate level of 18 plant positions within each experimental block.

Air temperature and relative humidity were recorded using four data loggers placed at canopy height in the four treatment areas (HOBO MX2300 series, Onset Computer Corporation, Bourne, MA, USA). Data was collected at 10−minute intervals throughout the experiment and during both temporal replications. The measured temperature was 22.38 ± 1.60 °C, and the relative humidity was 45.29 ± 7.05% (values are reported as average ± standard deviation). The EC and pH of the Hoagland’s solution used for irrigation were continuously controlled at 1.9 mS/cm and 6.5, respectively, by using an automatic controller (Bluelab Pro Controller Wi-Fi, Bluelab Corporation Limited, Tauranga, New Zealand) connected to a timer (Techbee Digital Timer outlet, DICP19, Techbee, China), starting irrigation twice a day.

### 2.3 Morphological measurements

**Figure 2:**
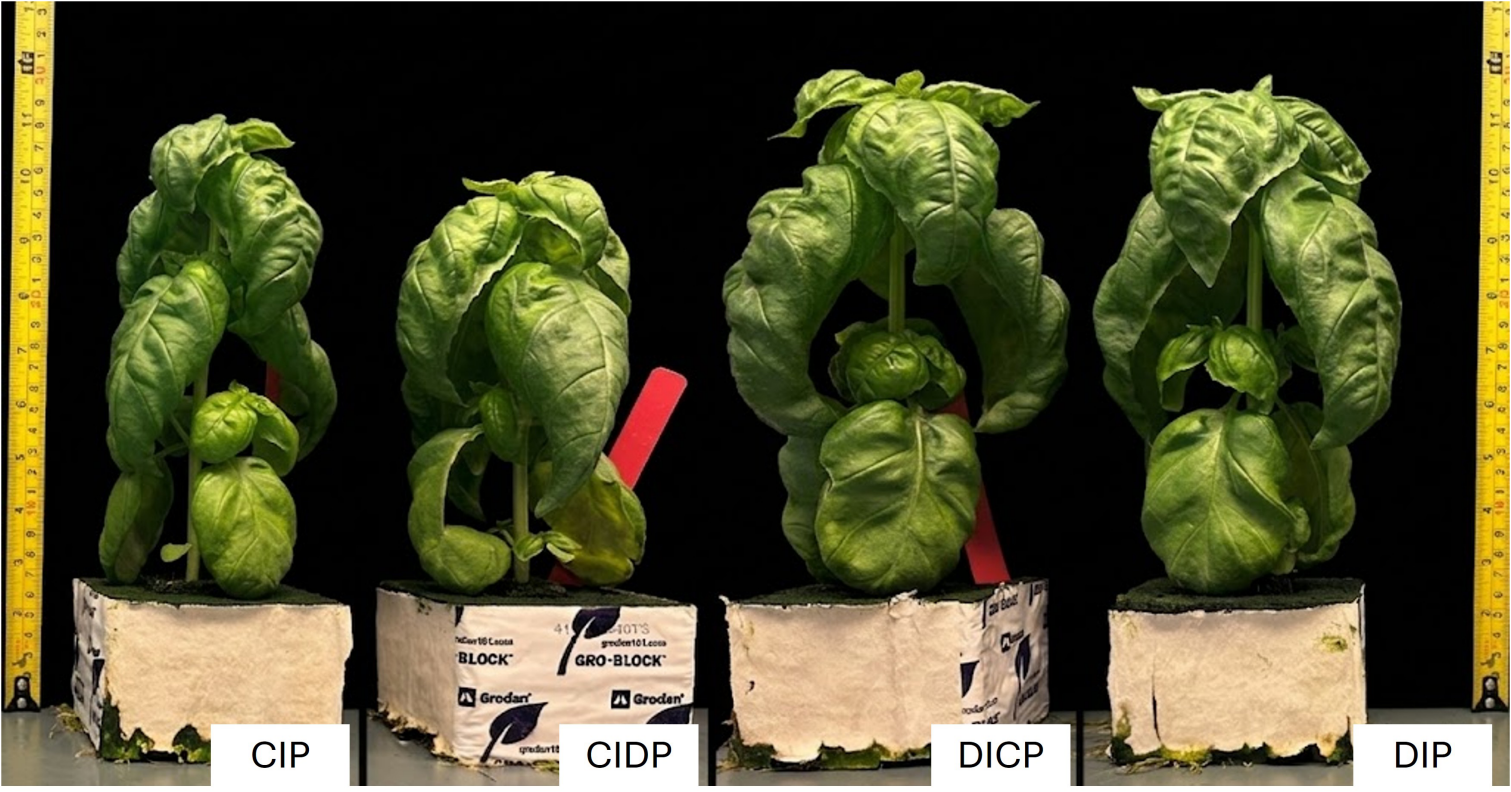
Representative morphology of basil (Ocimum basilicum L. cv. ‘Genovese’) plants at harvest stage (39 days after sowing) after being grown in climate-controlled conditions under varying light regimes (constant vs. gradually increasing light intensity and photoperiod) while maintaining equal daily light integral (DLI). Plants were cultivated in rockwool cubes; scale is provided by the lateral measurement tape.

Morphological parameters, including plant height, leaf area, fresh weight, dry weight, leaf number, and stem diameter, were considered for analysis of the impact of dynamic lighting treatments. Plant height was measured at the end of the experiment on day 39 from sowing (**Figure 2**). The eight plants, located in the center of each experimental block, served as measurement plants in each treatment to minimize border effects. The plants were harvested after a 24-day treatment period. The leaf area was measured by using a LI-3000C Leaf Area Meter (LI-COR Biosciences, Lincoln, Nebraska, USA). The fresh weight of each plant was measured separately for leaves and stems using an analytical balance (OHAUS, CR 621, China). The harvested plants were dried in an oven at 70 °C for 72 hours to determine their dry weight. The number of leaves was counted manually, and the stem diameter was measured with a Vernier caliper. The specific leaf area, specific stem length, and biomass partitioning traits, including stem dry weight fraction and leaf dry weight fraction, were estimated from the measured data using Equations 1, 2, and 3 reported below (Chen et al., 2022).

(1) $$\text{Specific leaf area}=\frac{\text{leaf area}\left({\text{cm}}^{2}\right)}{\text{leaf dry weight}\left(\text{g}\right)}\text{… … … … … …}$$

(2) $$\text{Specific stem length}=\frac{\text{Plant height}\left(\text{cm}\right)}{\text{Stem dry weight}\left(\text{g}\right)}\text{… … … … …}$$

(3) $$\text{Stem or leaf biomass allocation}=\frac{\text{Stem or leaf dry weight per plant }(\text{g})}{\text{Total shoot dry weight per plant }(\text{g}))}\times 100\%\text{…}$$

### 2.4 Physiological measurements

In this study, plant physiological performance was assessed by measuring stomatal conductance, destructive and non-destructive chlorophyll content, chlorophyll fluorescence, and LUE was calculated.

#### 2.4.1 Stomatal conductance

Stomatal conductance describes the degree of stomatal opening and reflects the diffusion of water vapor out of the leaf and CO_2_ into the leaf through the stomata, thereby influencing transpiration and gas exchange. It was measured once during the experiment at 38 days after sowing using a handheld porometer (LI-600, LI-COR Biosciences, Lincoln, NE, USA). Measurements were taken between 10:00 AM and 12:00 PM from the abaxial surface of the fully expanded third and fourth leaves counted from the apex of each plant, representing mature upper-canopy leaves of similar age and size. For each light treatment, measurements were averaged from eight leaves collected from four randomly selected plants.

#### 2.4.2 Destructive pigment content

At the last harvest, 39 days after planting, leaf samples were taken. Only fully developed leaves were picked, flash-frozen in liquid nitrogen to stop metabolic activity and stored at -80 °C until further analysis. Before being chemically tested, samples were finely ground with liquid nitrogen. An ethanol-based method was used to extract chlorophyll and carotenoids. About 0.030 g of ground tissue was weighed into test tubes and extracted with 2 mL of 95% (v/v) ethanol in a fume hood under low light conditions. Samples were vortexed, wrapped in aluminum foil to prevent degradation in the light, and stored at approximately 4 °C for 24 hours. After incubation, the samples were spun at 2500 rpm for 10 minutes, and the supernatant was moved to new tubes. The extraction step was repeated on the remaining pellet with 2 mL of solvent, and the samples were then centrifuged. The supernatants were then mixed together for each sample. Using a UV-Vis spectrophotometer, we measured the absorbance at 470, 648.6, and 664.1 nm for 170 µL samples of the extract pipetted into a half-area 96-well plate, ensuring a 1-cm path length. Amounts of chlorophyll a, chlorophyll b, and total carotenoids were calculated following the procedure described in Cammarisano et al. (2021).

#### 2.4.3 Non-destructive chlorophyll content

Relative chlorophyll content was measured one day before harvest, around 12:00-3:00 PM, using a chlorophyll content meter (Model: CCI-200, Opti-Sciences Inc., USA) to minimize diurnal variability in readings and to capture cumulative treatment effects on chlorophyll at harvest. Measurements were taken from the adaxial surface of the fully expanded third leaf from the top (Akter et al., 2024). For each treatment, readings from eight leaves on eight different plants were averaged.

#### 2.4.4 Chlorophyll fluorescence

Chlorophyll a fluorescence was measured using a portable pulse-modulated fluorometer (Model: OS5p+, Opti-Sciences Inc., USA) at three time points before each treatment change. For the first measurement, the two fully expanded bottom leaves were used. For the second and third measurements, the third and fourth fully expanded leaves from the top were selected. Light-adapted measurements (Fv′/Fm′) were recorded first, which represent the operating efficiency of Photosystem II under light conditions, followed by the maximum quantum yield of primary photosystem II photochemistry in dark adaptation (Fv/Fm) of the same leaves for 30 minutes using dark-adaptation leaf clips (Kalaji et al., 2017). Non-photochemical quenching (NPQ) was calculated as in Equation 4 below.

(4) $$\text{NPQ}=\frac{\left(\text{Fm}-{\text{Fm}}^{\prime }\right)}{{\text{Fm}}^{\prime }}\text{… … … … …}$$

where Fm is the maximal fluorescence in the dark-adapted state and Fm′ is the maximal fluorescence in the light-adapted state. The fluorometer utilizes a modulated light source and saturating pulses to measure fluorescence parameters via an optical fiber. The measurement protocol described in (Cammarisano et al., 2021) was followed for fluorescence measurements.

#### 2.4.5 Light use efficiency

Light use efficiency (LUE) is a critical parameter that quantifies how effectively a plant converts light (typically within the extended photosynthetically active radiation, ePAR, region) into biomass or yield. LUE was calculated as in (Larsen et al., 2020) adopting the following Equation 5.

(5) $$\text{LUE}\left(\text{g mo}{\text{l}}^{-1}400-800\text{ nm}\right)=\frac{\text{Plant dry weight}\left(\text{g}\right)\times \text{Plant density}\left(\text{plants }{\text{m}}^{-2}\right)}{\left(\text{Daily Light Integral }400-800\text{ nm}\left(\text{mol }{\text{m}}^{-2}{\text{d}}^{-1}\right)\times \text{Days of cultivation}\left(\text{d}\right)\right)}\ldots$$

In this study, LUE represents the efficiency of converting supplied PAR into plant dry biomass under controlled environment conditions. The denominator reflects the cumulative incident DLI supplied to the crop during the treatment period rather than absorbed or intercepted light at the canopy level. Therefore, the calculated LUE should be interpreted as a production-based efficiency metric that describes biomass accumulation per unit of light energy supplied.

### 2.5 Statistical analysis

All collected data were analyzed using one-way ANOVA, followed by Tukey’s HSD test to determine significant differences between treatments at (*P* ≤ 0.05). Statistical analyses were conducted in RStudio using several packages, including readr (Wickham et al., 2024), dplyr (Wickham et al., 2023), ggplot2 (Wickham, 2016), agricolae (de Mendiburu, 2023), and corrplot (Wei and Simko, 2021), within the R statistical environment (R Core Team, 2024). The experiment was replicated twice in time. For morphological and non-destructive chlorophyll measurements, eight plants were sampled within each treatment area during each temporal replication. These plants were treated as biological sub-samples within each independently repeated experimental run. Measurements of Fv/Fm and Fv′/Fm′ were conducted on three plants per replicate, while stomatal conductance measurements were conducted on four plants per replicate. For destructive chlorophyll analysis, each treatment comprised six biological replicates (three per temporal replication of the study), with each biological replicate including three technical replicates. In addition, relationships among morphological traits were evaluated using Pearson correlation analysis. A correlation matrix was generated from selected morphological variables and visualized as a heatmap using the corrplot package in R (Wei and Simko, 2021). The heatmap was constructed to illustrate the strength and direction of pairwise correlations, with color gradients indicating negative-to-positive associations. Pearson correlation analysis was performed using pooled morphological observations across all treatments and temporal replications (n = 64 observations). Correlation coefficients were tested for statistical significance at P ≤ 0.05.

## 3 Results

### 3.1 Effects of dynamic lighting on morphology and biomass

**Figure 3:**
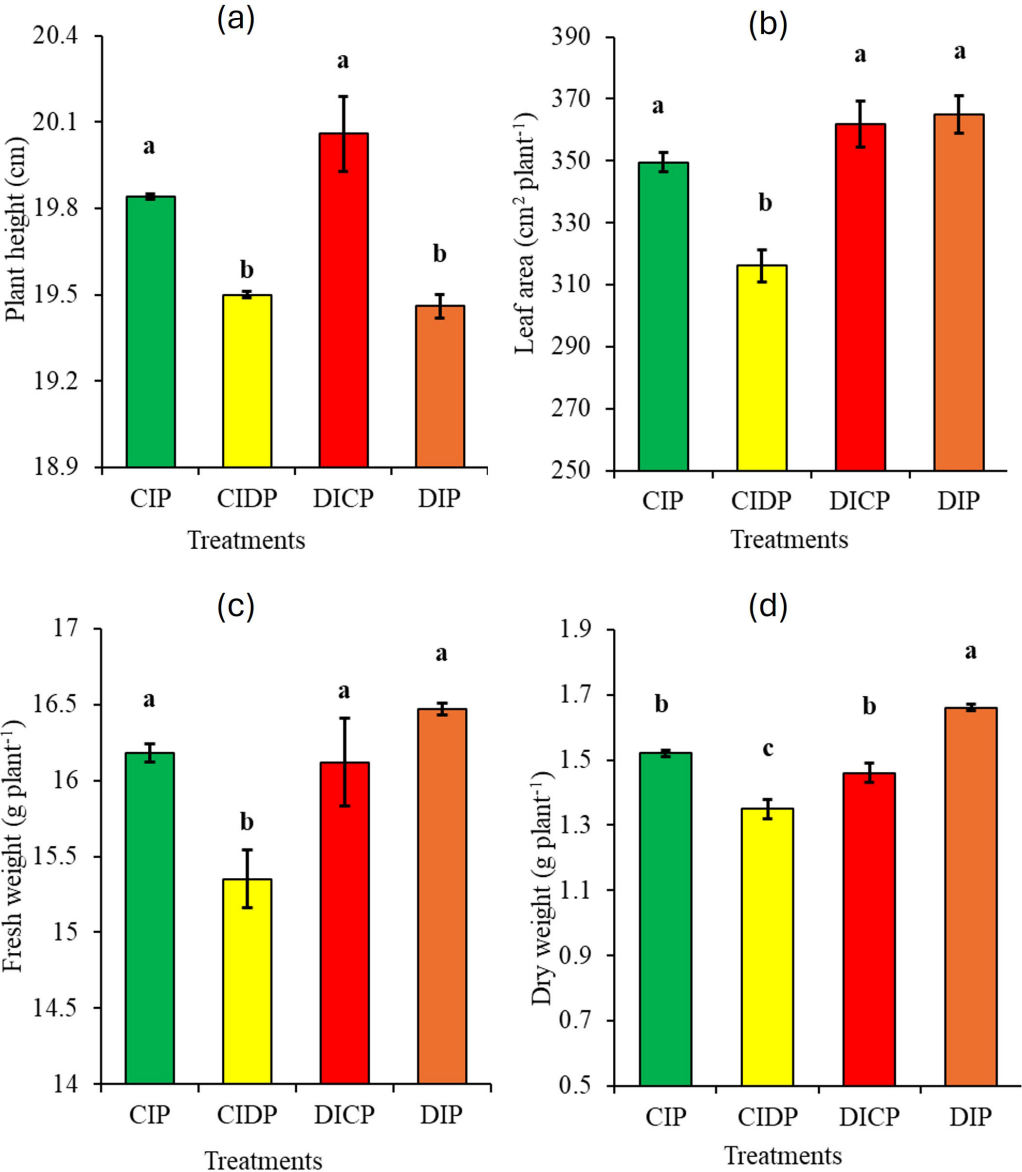
Bar graphs showing plant height **(a)**, leaf area **(b)**, fresh weight **(c)**, and dry weight **(d)** of basil plants treated with four light regimes CIP, Constant Intensity and Photoperiod; CIDP, Constant Intensity, Dynamic Photoperiod; DICP, Dynamic Intensity, Constant Photoperiod; DIP, Dynamic Intensity and Photoperiod. Measurements were conducted at harvest, 39 days after sowing and 24 days after treatments began. Values represent the mean ± standard error (SE) of two temporal replicates (independent experimental runs) per treatment. Different letters above bars indicate significant differences at p ≤ 0.05.

A gradual increase in PFD and photoperiod over eight days significantly affected the morphology and biomass of the basil plants (**Figure 3**; **Tables 3**, **4**). **Figure 3** shows the impact of dynamic lighting treatments on plant height, leaf area, fresh weight, and dry weight. Plant height varied significantly among treatments (*P* ≤ 0.01). The tallest plants were recorded under DICP (20.06 cm), followed closely by CIP (19.84 cm), both of which were significantly taller than CIDP (19.50 cm) and DIP (19.46 cm), which formed a separate statistical group (**Figure 3A**). Significant differences in total leaf area were observed among treatments (*P* ≤ 0.05). DIP (364 cm²), DICP (361 cm²), and CIP (349 cm²) formed the top group, with no statistical differences among them; all outperformed CIDP (316 cm²) (**Figure 3B**). Fresh weight differed significantly across treatments (*P* ≤ 0.05), with DIP producing the highest yield (16.47 g plant^-^¹), followed by CIP (16.18 g plant^-^¹) and DICP (16.12 g plant^-^¹), while CIDP had the lowest value (15.35 g plant^-^¹) (**Figure 3C**). Dry weight also varied significantly (*P* ≤ 0.001), with DIP again recording the highest value (1.66 g plant^-^¹) and CIDP the lowest (1.35 g plant^-^¹). CIP and DICP were intermediate (**Figure 3D**).

**Table 3:** Effects of four light treatments CIP, constant Intensity and photoperiod; CIDP, constant intensity, dynamic photoperiod; DICP, dynamic intensity, constant photoperiod; DIP, dynamic intensity and photoperiod on basil leaf number, stem diameter, dry matter content (DMC), stem dry weight (DW), and leaf DW.

Treatment	Leaf number	Stem diameter (mm)	DMC (%)	Stem DW (g plant^-1^)	Leaf DW (g plant^-1^)
CIP	13.10 ± 0.03 b	4.87 ± 0.02 b	8.66 ± 0.01 d	0.29 ± 0.01 a	1.24 ± 0.02 b
CIDP	12.25 ± 0.45 b	4.78 ± 0.01 c	9.17 ± 0.03 b	0.25 ± 0.00 b	1.09 ± 0.02 c
DICP	14.80 ± 0.27 a	5.05 ± 0.03 a	8.99 ± 0.03 c	0.24 ± 0.01 b	1.21 ± 0.02 b
DIP	12.87 ± 0.34 b	5.13 ± 0.02 a	9.93 ± 0.01 a	0.29 ± 0.00 a	1.37 ± 0.01 a
P value	0.017	< 0.001	< 0.001	<0.001	0.001
LSD	1.223	0.077	0.104	0.021	0.063

Measurements were conducted at harvest, 39 days after sowing and 24 days after treatments began.

Values are means ± SE (n = 2). Different letters within a row indicate significant differences according to the LSD test at *P* ≤ 0.05. Each replicate (n) comprises 8 individual plants.

**Table 4:** Effects of four light treatments (CIP, constant intensity and photoperiod; CIDP, constant intensity, dynamic photoperiod; DICP, dynamic intensity, constant photoperiod; DIP, dynamic intensity and photoperiod) on specific leaf area (SLA), specific stem length (SSL), stem leaf ratio, stem dry weight fraction (DWF), and leaf DWF.

Treatment	Specific leaf area (cm² g^-1^)	Specific stem length (cm g_-1_)	Stem leaf ratio	Stem DWF (%)	Leaf DWF (%)
CIP	300.89 ± 0.99 b	73.06 ± 1.00 d	0.24 ± 0.01 a	18.69 ± 0.32 a	81.32 ± 0.32 c
CIDP	266.68 ± 2.37 d	87.69 ± 1.08 b	0.23 ± 0.00 a	18.61 ± 0.05 a	81.39 ± 0.05 c
DICP	310.64 ± 1.48 a	93.24 ± 1.34 a	0.20 ± 0.00 c	16.32 ± 0.13 c	83.68 ± 0.13 a
DIP	288.86 ± 1.30 c	79.50 ± 0.33 c	0.22 ± 0.01 b	17.44 ± 0.08 b	82.56 ± 0.08 b
P value	< 0.001	< 0.001	< 0.001	0.002	0.002
LSD	6.347	3.955	0.014	0.694	0.694

Measurements were conducted on basil plants at harvest, 39 days after sowing and 24 days after treatments began.

Values are means ± SE (n = 2). Different letters within a row indicate significant differences according to the LSD test at *P* ≤ 0.05. Each replicate comprised 8 individual plants.

Other parameters, including leaf number and stem diameters, along with estimated parameters such as dry matter content (DMC), stem dry weight, and leaf dry weight, are presented in **Table 3**. The number of leaves was significantly affected by the treatments (*P* ≤ 0.05). Plants under DICP exhibited the highest leaf number (14), which was significantly greater than that of all other treatments. CIP, CIDP, and DIP (12) did not differ significantly from each other. Stem thickness was influenced significantly by the treatments (*P* ≤ 0.001). DIP and DICP recorded the greatest stem diameters (5.13 mm and 5.05 mm, respectively), followed by CIP (4.87 mm), while CIDP exhibited the lowest value (4.78 mm). DMC differed significantly among treatments (P ≤ 0.001). The highest DMC was recorded under DIP (9.93%), followed by CIDP (9.17%), DICP (8.99%), and CIP (8.66%). Stem dry weight was highest in CIP and DIP (0.29 g), significantly greater than CIDP and DICP (P ≤ 0.01). Leaf dry weight differed significantly among treatments (*P* ≤ 0.001). DIP had the highest mean (1.37 g plant^-^¹), significantly greater than CIP (1.24 g plant^-^¹) and DICP (1.21 g plant^-^¹), which did not differ from each other, while CIDP (1.09 g plant^-^¹) had the lowest value.

**Table 4** presents the specific leaf area (SLA), specific stem length (SSL), stem-leaf ratio, stem dry weight fraction (DWF), and leaf DWF. SLA differed significantly among treatments (*P* ≤ 0.001). DICP had the highest SLA (310 cm² g^-^¹), followed by CIP, DIP, and the lowest in CIDP (266.68 cm² g^-^¹). SSL also differed (*P* ≤ 0.001), with DICP showing the greatest elongation (93.24 cm g^-^¹) and CIP the shortest (73 cm g^-^¹). The stem-to-leaf ratio was significantly lower in DICP (0.20) compared to others (*P* ≤ 0.001). CIP and CIDP had higher ratios (0.24 and 0.23, respectively), indicating greater stem biomass. Stem and leaf DWF also varied significantly (*P* ≤ 0.01). CIP showed the highest stem DWF (18.69), while DICP had the lowest (16.32). Leaf DWF followed a similar trend, with the highest value in DICP (83.68) and the lowest in CIP (81.32).

#### 3.1.1 Pearson correlation heatmap of morphological and biomass traits

**Figure 4:**
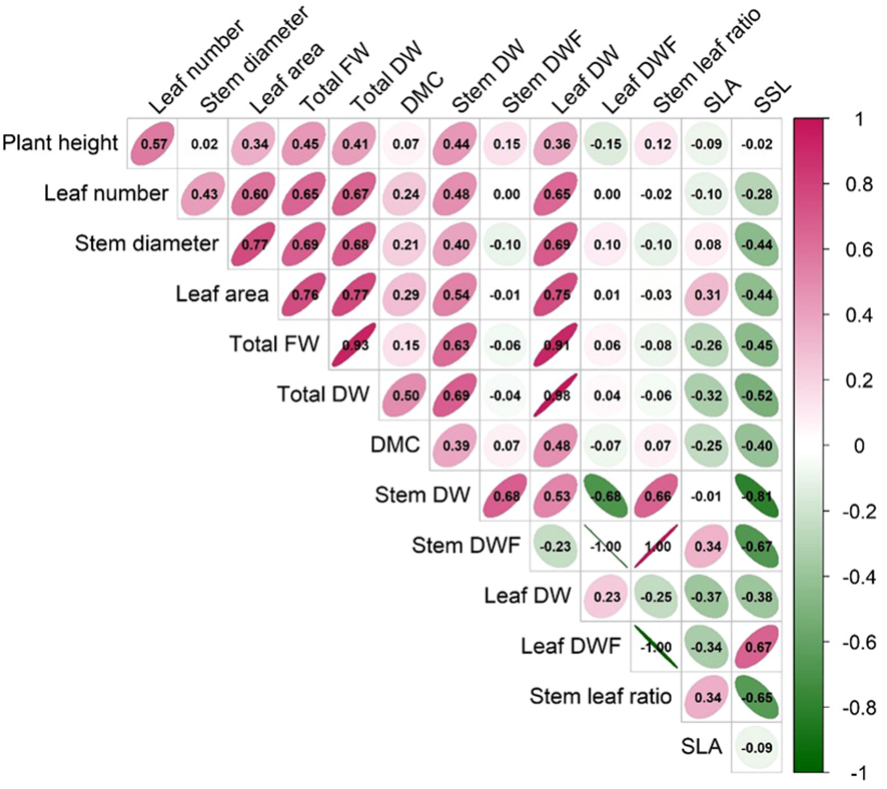
Pearson correlation heatmap illustrates relationships among morphological and biomass-related traits of basil plants across all treatments. The color gradient represents the strength and direction of correlations, ranging from strong negative (green, -1) to strong positive (pink, +1), with white indicating no correlation. Ellipse shape and orientation further reflect the magnitude and direction of the correlation. Values within cells represent Pearson correlation coefficients. Fresh weight (FW); dry weight (DW); dry matter content (DMC); specific leaf area (SLA); specific stem length (SSL).

Pearson correlation heatmap showing the relationships among morphological and biomass-related traits in basil plants across all treatments. Strong positive correlations were observed among key growth parameters, indicating coordinated plant development. For instance, leaf number was positively correlated with total fresh weight (*r* = 0.65), whereas stem diameter was strongly associated with leaf area (*r* = 0.77). Leaf area was also strongly correlated with total fresh weight (*r* = 0.76), highlighting its contribution to biomass accumulation. A very strong positive correlation was observed between total fresh weight and total dry weight (*r* = 0.93), reflecting consistent biomass production. Additionally, total dry weight was moderately correlated with dry matter content (*r* = 0.50). In contrast, several negative relationships were identified. Stem dry weight showed a strong negative correlation with leaf DWF (r = −0.68). In addition, stem DWF exhibited a perfect negative correlation with leaf DWF (r = −1.00), reflecting their complementary calculation from total shoot dry weight rather than an independent biological relationship. Furthermore, SSL was negatively correlated with total dry weight (*r* = −0.52) and stem leaf ratio (*r* = −0.65) (**Figure 4**).

### 3.2 Effects of dynamic lighting on physiological responses

**Figure 5:**
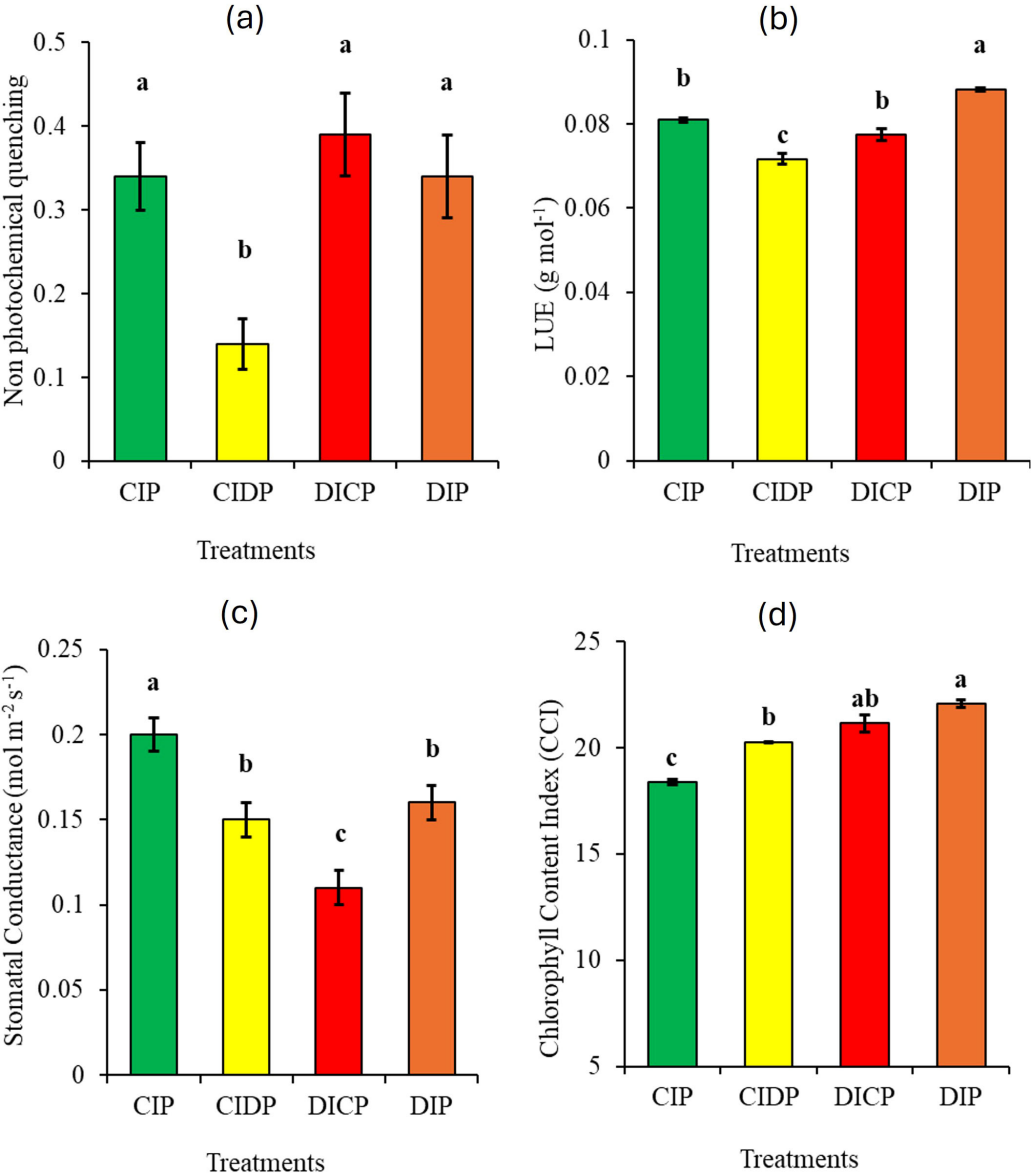
Effects of four light treatments (CIP; Constant Intensity and Photoperiod, CIDP; Constant Intensity, Dynamic Photoperiod, DICP; Dynamic Intensity, Constant Photoperiod, DIP; Dynamic Intensity and Photoperiod) on non-photochemical quenching (NPQ) **(a)**, Light Use Efficiency (LUE) **(b)**, stomatal conductance **(c)**, and non-destructive chlorophyll **(d)** in basil plants. Measurements were conducted on basil plants at harvest, 39 days after sowing and 24 days after treatments began. Values represent the mean ± standard error (SE) of two temporal replicates (independent experimental runs) per treatment. Different letters above bars indicate significant differences at p ≤ 0.05. a denotes non-significant differences.

**Figure 5** shows non-photochemical quenching (NPQ), LUE, stomatal conductance, and chlorophyll content index (CCI). NPQ was significantly influenced by the treatments (*P* ≤ 0.001); the highest NPQ was recorded in DICP (0.39), followed by CIP and DIP (both 0.34), all of which were significantly higher than CIDP (0.14) (**Figure 5A**). LUE varied significantly among treatments (*P* ≤ 0.001). DIP showed the highest LUE value (0.088 g mol^-^¹), significantly greater than all other treatments. CIP (0.081 g mol^-^¹) and DICP (0.077 g mol^-^¹) were statistically similar and exhibited intermediate LUE values. CIDP showed the lowest LUE (0.072 g mol^-^¹) (**Figure 5B**). Stomatal conductance showed significant differences among treatments (*P* ≤ 0.001). CIP exhibited the highest stomatal conductance (0.20 mol m^-^² s^-^¹), while DICP had the lowest value (0.11 mol m^-^² s^-^¹), with CIDP and DIP falling in between CIP and DICP, but statistically identical among them (**Figure 5C**). Non-destructive chlorophyll content (CCI) differed significantly among treatments (*P* ≤ 0.001), with the highest value recorded under DIP (22.06 units), followed by DICP (21.16) and CIDP (20.28), while CIP had the lowest relative chlorophyll content (18.39) (**Figure 5D**).

#### 3.2.1 Chlorophyll a fluorescence parameters

**Supplementary Table 1** presents the chlorophyll a fluorescence parameter of the plant under various dynamic lighting treatments at different growth stages. Chlorophyll fluorescence parameters were generally not significantly affected by the treatments. However, significant differences were observed in Fv′/Fm′ (light) and Fv/Fm (dark) during the second week of measurement (P ≤ 0.05). During this period, DICP exhibited the highest Fv′/Fm′ value (0.747), while CIP had the lowest (0.720). Treatments CIDP (0.734) and DIP (0.729) showed intermediate values and were not significantly different from each other. Similarly, Fv/Fm (dark) was higher in CIP and DICP (0.806-0.805) compared to CIDP and DIP (0.789-0.795); however, the difference was not statistically significant, except during the second week of the growing stage.

#### 3.2.2 Destructive pigments

**Table 5** represents the destructive chlorophyll analysis, which revealed significant effects of lighting treatments on pigment composition in basil plants. Significant differences were observed for chlorophyll a, chlorophyll b, carotenoids, and total chlorophyll (a+b) (*P* ≤ 0.05), whereas the chlorophyll a/b ratio was not significantly affected by the treatments. For chlorophyll a, CIP (1.051 mg g^-1^ of FW), DICP (1.001 mg g^-1^ of FW), and DIP (1.052 mg g^-1^ of FW) exhibited significantly higher values compared to CIDP (0.843 mg g^-1^ of FW). A similar trend was observed for chlorophyll b, where CIP (0.293 mg g^-1^ of FW), DICP (0.268 mg g^-1^ of FW), and DIP (0.285 mg g^-1^ of FW) showed higher concentrations than CIDP (0.224 mg g^-1^ of FW). In terms of carotenoids, CIP (0.262 mg g^-1^ of FW), DICP (0.255 mg g^-1^ of FW), and DIP (0.265 mg g^-1^ of FW) had significantly greater values compared to CIDP (0.202 mg g^-1^ of FW). Consequently, total chlorophyll (chlorophyll a+b) followed the same pattern, with higher values in CIP (1.344 mg g^-1^ of FW), DICP (1.269 mg g^-1^ of FW), and DIP (1.337 mg g^-1^ of FW) relative to CIDP (1.067 mg g^-1^ of FW).

**Table 5:** Chlorophyll and carotenoid content of basil plants grown under four dynamic lighting treatments (CIP, constant intensity and photoperiod; CIDP, constant intensity, dynamic photoperiod; DICP, dynamic intensity, constant photoperiod; DIP, dynamic intensity and photoperiod).

Treatments	Chlorophyll a (mg g^-1^FW)	Chlorophyll b (mg g^-1^FW)	Carotenoids (mg g^-1^FW)	Chlorophyll a/b	Chlorophyll a+b (mg g^-1^FW)
CIP	1.051 ± 0.052 a	0.293 ± 0.012 a	0.262 ± 0.016 a	3.579 ± 0.047a	1.344 ± 0.064 a
CIDP	0.843 ± 0.046 b	0.224 ± 0.010 b	0.202 ± 0.009 b	3.761 ± 0.064a	1.067 ± 0.055 b
DICP	1.001 ± 0.068 a	0.268 ± 0.021 a	0.255 ± 0.025 a	3.749 ± 0.035a	1.269 ± 0.088 a
DIP	1.052 ± 0.037 a	0.285 ± 0.013 a	0.265 ± 0.009 a	3.706 ± 0.047a	1.337 ± 0.050 a
P value	0.0365	0.0173	0.0398	0.0692	0.0311
LSD	0.1555	0.0437	0.0478	0.1477	0.1984

Basil leaf samples were collected at harvest, 39 days after sowing and 24 days after treatments began.

Values are means ± SE. Different letters within a column indicate significant differences at *P* ≤ 0.05 based on the LSD test; the same letter indicates no significant difference. Each treatment comprised six biological replicates (three per temporal replication of the study), with each biological replicate including three technical replicates.

Although the chlorophyll a/b ratio showed some variation among treatments, with CIDP (3.761) and DICP (3.749) tending to be higher than CIP (3.579), these differences were not statistically significant (*P* ≥ 0.0692). Overall, the results indicate that CIDP consistently resulted in lower pigment concentrations, while CIP, DICP, and DIP maintained higher levels of chlorophyll and carotenoids, suggesting a stronger photosynthetic pigment capacity under these treatments.

## 4 Discussion

### 4.1 Gradual increases in light intensity and photoperiod enhance dry matter accumulation and LUE

This study evaluated how gradual increases in light intensity and photoperiod, applied either simultaneously or independently, while maintaining a constant DLI affected growth, dry matter accumulation, and LUE in basil. It was hypothesized that a dynamic lighting regime would better match plant developmental needs and improve biomass production and LUE. The PFD levels used in this study (200-400 μmol m^-^² s^-^¹) were selected to represent moderate-to-high lighting conditions commonly used for indoor basil production under controlled environment systems (Dou et al., 2018; Larsen et al., 2020). These light levels were intended to avoid both light limitation and excessive photoinhibitory conditions while allowing evaluation of dynamic lighting responses across developmental stages. Previous studies have shown that plant light requirements vary across developmental stages, with younger plants exhibiting lower light demand due to limited photosynthetic capacity, while mature plants can utilize higher light intensities more effectively (Eghbal et al., 2024; Stagnari et al., 2018). In the present study, although leaf area and fresh weight did not differ significantly between the fully dynamic treatment (DIP) and the constant light control (CIP), dry biomass accumulation was significantly higher (by 9%) in DIP (**Figures 3B–D**). This increase in dry biomass was accompanied by a 9% improvement in LUE, indicating that plants under dynamic lighting were more efficient in converting light into biomass. Because LUE in this study was calculated based on supplied DLI rather than absorbed light or direct photosynthetic measurements, the observed increase should be interpreted as improved biomass production efficiency under the applied lighting regime. The higher LUE observed under DIP treatment suggests more efficient biomass accumulation per unit of supplied light. These findings suggest that optimizing the temporal distribution of light, rather than increasing total DLI, can enhance biomass production efficiency in basil. Similar responses were reported by Larsen et al. (2020), who found that LUE based on dry mass increased with increasing photosynthetic photon flux density (PPFD), even when fresh mass responses were limited. In their study, PPFD ranged from 50 to 600 µmol m^-^² s^-^¹ during end-of-production treatments, and LUE calculations incorporated plant dry mass, plant density, DLI, and cultivation duration, emphasizing the importance of integrating both biomass production and light input over time. This aligns with our approach of maintaining constant DLI while dynamically adjusting light intensity and photoperiod, demonstrating that temporal light optimization can improve LUE and dry matter accumulation in basil. Furthermore, adaptive lighting strategies have been proposed to enhance LUE by aligning light supply with photosynthetic capacity and promoting acclimation in controlled environments (Neo et al., 2022; Paponov and Paponov, 2025; Ramanna et al., 2017).

The enhanced dry matter accumulation and LUE observed under DIP treatment were likely driven by physiological and structural adjustments rather than differences in overall plant size. The absence of significant differences in fresh biomass and leaf area among treatments suggests that the benefits of dynamic lighting were not due to increased canopy expansion, but rather more efficient biomass accumulation and allocation under the supplied light conditions. Under DIP treatment, the gradual increase in light intensity and extended photoperiod were associated with higher chlorophyll content index (CCI) and structural changes in leaves, including reduced SLA, indicating thicker or denser leaves. Reduced SLA generally reflects greater structural investment per unit leaf area and may be associated with increased mesophyll development or enhanced accumulation of structural tissues under dynamic lighting conditions (Larsen et al., 2020; Pennisi et al., 2020). Notably, leaf dry weight increased under DIP without a corresponding change in leaf dry weight fraction, suggesting that the treatment promoted greater biomass accumulation without substantially altering biomass partitioning patterns. These findings suggest that dynamic lighting may have promoted more efficient biomass accumulation per unit leaf area while maintaining relatively stable tissue composition. Although leaf texture and postharvest quality were not directly evaluated, such structural changes may have implications for basil handling, drying efficiency, and suitability for fresh versus processing applications.

Higher chlorophyll content, reduced SLA, and increased leaf dry weight are known to enhance photosynthetic capacity and contribute to increased dry matter accumulation (Dou et al., 2019; Moratiel et al., 2023; Proietti et al., 2023). In contrast to other dynamic treatments (CIDP and DICP), DIP treatment involved a coordinated adjustment of both light intensity and photoperiod, likely allowing plants to adjust more gradually to daily light conditions and maintain more stable photosynthetic function. Such acclimation may allow plants to maintain stable physiological performance under gradually changing light conditions. Similar responses have been reported in other plant species, in which dynamic lighting strategies promoted denser tissue development and improved resource use efficiency (Larsen et al., 2020; Pennisi et al., 2020). However, because direct measurements of gas exchange or carbon fixation were not conducted, interpretations of photosynthetic performance and carbon assimilation remain tentative.

Overall, these findings support the concept that dynamic light regimes designed to mimic the natural progression of plant light demand can enhance LUE and dry biomass production in basil under CEA systems. From an applied perspective, increased dry matter accumulation under dynamic lighting may be advantageous for the basil processing industry, as higher dry weight can improve drying efficiency and final product yield. This suggests that adaptive lighting strategies can optimize production outcomes without increasing total DLI, offering a promising approach for indoor cultivation systems.

### 4.2 Dynamic lighting affects chlorophyll content and stomatal conductance

In this study, dynamic light regimes were hypothesized to enhance physiological performance in basil. Several physiological processes, such as the quantum efficiency of PSII, stomatal conductance, and chlorophyll accumulation, are crucial determinants of plant productivity. Chlorophyll fluorescence parameters, including Fv/Fm (Maximum quantum yield of primary photosystem II photochemistry) and Fv′/Fm′ (Operating efficiency of Photosystem II in light), showed no significant differences among all treatments (**Supplementary Table 1**). The maximum quantum yield of PSII (Fv/Fm) remained unaffected across treatments, suggesting minimal photoinhibition and effective energy conversion under all light conditions tested, with all light treatments likely providing optimal light for basil growth (Kabacheuskaya et al., 2024). All treatments maintained Fv/Fm values near 0.83, indicating that the photosynthetic apparatus in basil was functioning efficiently. Values around 0.83 are typically characteristic of healthy, well-functioning PSII in the absence of stress. This aligns with findings by Fu et al. (2012), who reported that lettuce maintained stable and high Fv/Fm values under 200, 400, and 600 µmol m^-2^ s^-1^ light, and low values at 100 and 800 µmol m^-2^ s^-2^, suggesting that such light conditions support optimal photosystem II efficiency. Similarly, NPQ, a key mechanism for dissipating excess light, was not statistically different between CIP and DIP (**Figure 5A**). These observations support our results and suggest that the photosynthetic apparatus in basil is relatively resilient to gradual temporal changes in light, provided the total DLI (17 mol m^-^² day^-^¹, average light intensity of 300 µmol m^-^² s^-^¹ and a 16 photoperiod) remains within the optimal range. Similarly Larsen et al. (2022) demonstrated that basil growth and quality improved as light intensity increased up to an optimal range of 250-290 µmol m^-^² s^-^¹. Their findings further suggested that basil can acclimate to moderate-to-high light environments without substantial photoinhibitory stress or impairment of photoprotective function. However, CIDP exhibited a substantially lower NPQ than CIP, despite both treatments receiving similar light intensity. This response suggests that the gradual extension of photoperiod alone may have influenced photoprotective acclimation and energy-dissipation mechanisms independently of PFD. Notably, CIDP also showed lower biomass accumulation, chlorophyll content, and LUE relative to the other treatments, suggesting that extending the photoperiod without increasing light intensity may limit the degree of physiological acclimation achievable under dynamic lighting. Although the underlying mechanisms remain unclear, these observations indicate that photoperiod extension alone has a reduced capacity to induce higher NPQ compared with treatments involving increased PFD.

In contrast to expectations, stomatal conductance was significantly higher (25%) under constant light (CIP) than under dynamic light treatments (CIDP, DICP, and DIP) (**Figure 5C**). Higher stomatal conductance under constant lighting may reflect more stable irradiance conditions that promoted sustained stomatal opening and transpiration throughout the photoperiod (Matthews et al., 2020; Vialet-Chabrand et al., 2017). In contrast, the lower stomatal conductance observed under dynamic lighting treatments may indicate physiological acclimation to gradually changing light environments, potentially contributing to a more conservative balance between water loss and biomass accumulation (Kaiser et al., 2015; Morales and Kaiser, 2020). Notably, extending photoperiod appeared to reduce stomatal conductance more strongly than increasing light intensity, suggesting that extended daily light exposure may influence stomatal regulation independently of PFD (Matthews et al., 2020). Despite lower stomatal conductance, DIP treatment maintained higher dry biomass accumulation and LUE, indicating that increased biomass production under dynamic lighting was not necessarily associated with greater stomatal opening. Similar responses have been reported by Lee and Kim (2024),who observed reduced stomatal conductance in basil under higher light intensity during drought conditions. However, unlike their study, our experiment did not impose water limitation or alter CO_2_ conditions. Therefore, interpretations of transpiration dynamics and water-use responses should be interpreted with caution, as transpiration and gas exchange rates were not directly measured in the present study.

Changes in irradiance can affect chlorophyll levels by regulating light-sensitive metabolic pathways and modulating both biosynthesis and degradation (Chang et al., 2008; Dou et al., 2019). Dynamic lighting can modulate these pathways to stabilize chloroplast development, maintain photosystem integrity, and enhance overall pigment accumulation (Aliniaeifard, 2024). In our study, basil plants grown under fully dynamic light regime (DIP) exhibited significantly higher relative chlorophyll levels than those under constant light (CIP) (**Figure 5D**). This suggests that temporal variations in light intensity and photoperiod may have promoted pigment biosynthesis and enhanced photosynthetic capacity by better regulating light-dependent metabolic responses.

However, the destructive pigment analysis revealed a different pattern: chlorophyll and carotenoid contents were similar among CIP, DICP, and DIP, whereas CIDP treatment consistently showed lower pigment concentrations. This would suggest that dynamic lighting does not inherently increase pigment biosynthesis and that specific regimes, such as dynamic photoperiod alone (CIDP), may disrupt the balance between light duration and intensity, thereby limiting chlorophyll accumulation.

The discrepancy between non-destructive and destructive measurements may be attributed to the fact that non-destructive methods (e.g., CCI) estimate relative chlorophyll content, which is influenced by leaf optical properties and thickness, whereas destructive analysis provides a more direct quantification of pigment concentration (Cammarisano et al., 2022).

## 5 Conclusions

Efficient light management in indoor basil cultivation remains a significant challenge, particularly in balancing energy use and optimal plant growth. This study evaluated the impact of constant vs. progressively increasing light intensity and photoperiod, with the same DLI, on the morphological and physiological response of basil. Among the fully dynamic light treatments, DIP (Dynamic Intensity and Photoperiod) was the most efficient. Although DIP treatment did not induce a significant increase in plant height, leaf area, or fresh weight compared to constant lighting (CIP) treatment, it improved dry weight and LUE by 9% compared to CIP treatment. In addition, dynamic light treatments, particularly DIP treatment, increased relative chlorophyll content, which may have contributed to the greater dry matter accumulation. These findings highlight the potential of dynamic light delivery to improve resource use efficiency, notably by increasing dry biomass per mole of light. This trait is particularly valuable for basil grown for processing applications, including pesto, dried herbs, and dietary supplements, where dry matter content directly influences final product yield and quality. Future research should also investigate the impact of dynamic lighting on secondary metabolites, such as volatile organic compounds, to further optimize quality in indoor basil production. Additionally, evaluating how dynamic lighting influences thermal loads and energy consumption per unit of production (kWh kg^-^¹ dry weight) will be essential for assessing its practical viability.

## Data Availability

The raw data supporting the conclusions of this article will be made available by the authors, without undue reservation.
