# Supplementary material for: Gradual increases in light intensity and photoperiod enhance light use efficiency and dry matter in indoor basil
Source: Front Plant Sci. 2026 Jun 12;17:1861031. doi: 10.3389/fpls.2026.1861031 (PMC13305995; doi:10.3389/fpls.2026.1861031)
Supplement: Supplementary file 1 [file DataSheet1.docx]

**Gradual Increases in Light Intensity and Photoperiod Enhance Light Use Efficiency and Dry Matter in Indoor Basil**

Nazmin Akter^1^, Shamim Ahamed^2^*, Gail Taylor^3^, and Laura Cammarisano^1^*

**^1^**Department of Plant Sciences, University of California, Davis, USA

**^2^**Department of Biological and Agricultural Engineering, University of California, Davis, USA

**^3^**Department of Botany, University College of London, London, United Kingdom

**Corresponding author:*
lcammarisano@ucdavis.edu
mahamed@ucdavis.edu

**Supplementary material**

**Supplementary Table 1:** Effects of four light treatments (CIP; Constant Intensity and Photoperiod, CIDP; Constant Intensity and Dynamic Photoperiod, DICP; Dynamic Intensity and Constant Photoperiod, DIP; Dynamic Intensity and Photoperiod) on chlorophyll fluorescence parameters (Fv/Fm and Fv′/Fm′) of basil plants. Measurements were conducted at three time points during the treatment period.

| **Treatment** | **Fv′/Fm′**  **1st (n=5)** | **Fv′/Fm′**  **2nd (n=5)** | **Fv′/Fm′**  **3rd (n=9)** | **Fv/Fm**  **1st (n=5)** | **Fv/Fm**  **2nd (n=5)** | **Fv/Fm**  **3rd (n=9)** |
| --- | --- | --- | --- | --- | --- | --- |
| CIP | 0.725 ± 0.006 **a** | 0.720 ± 0.004 **c** | 0.712 ± 0.012 **a** | 0.791 ± 0.003 **a** | 0.806 ± 0.003 **a** | 0.810 ± 0.001 **a** |
| CIDP | 0.727 ± 0.007 **a** | 0.734 ± 0.004 **ab** | 0.712 ± 0.018 **a** | 0.782 ± 0.003 **a** | 0.789 ± 0.003 **b** | 0.808 ± 0.002 **a** |
| DICP | 0.738 ± 0.004 **a** | 0.747 ± 0.004 **a** | 0.718 ± 0.004 **a** | 0.795 ± 0.003 **a** | 0.805 ± 0.003 **c** | 0.809 ± 0.001 **a** |
| DIP | 0.730 ± 0.006 **a** | 0.729 ± 0.004 **bc** | 0.711 ± 0.006 **a** | 0.788 ± 0.003 **a** | 0.795 ± 0.003 **b** | 0.807 ± 0.001 **a** |
| **P value** | 0.489 | 0.0053 | 0.974 | 0.051 | 0.0052 | 0.765 |
| **LSD value** | 0.0186 | 0.0137 | – | 0.0092 | 0.0100 | – |

**Note:** Values are means ± SE. Different letters within a column indicate significant differences at P ≤ 0.05 based on the LSD test; the same letter represents a non-significant difference. Here, Fv/Fm represents the maximum quantum efficiency of Photosystem II (PSII) in dark-adapted and Fv′/Fm′ represents the operating quantum efficiency of Photosystem II (PSII) in light.


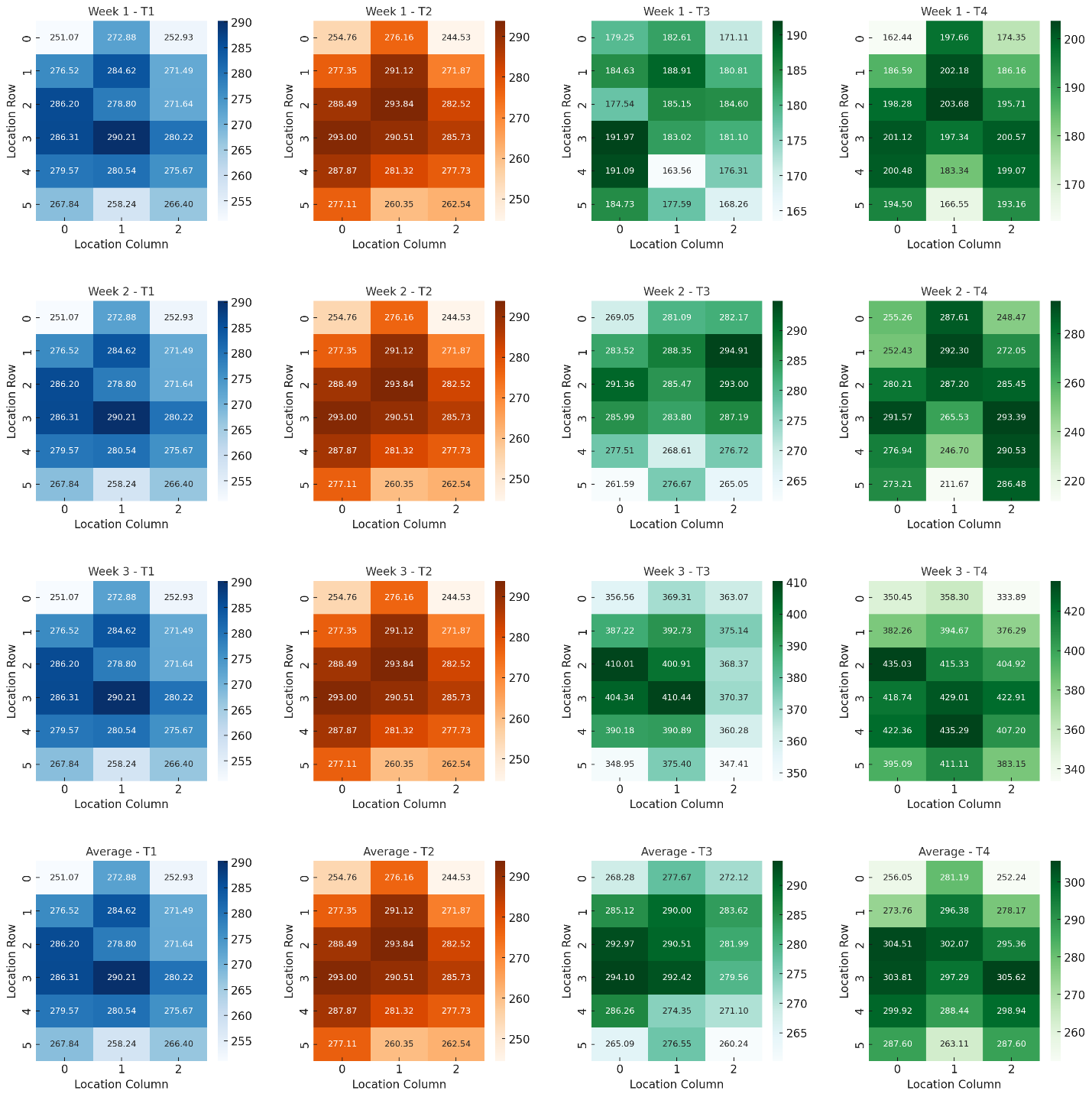


**Supplementary Figure 1.** Average spatial distribution of photon flux density (PFD) measured at 18 locations under four lighting treatments over the experimental period. Treatments are arranged from left to right as: T1 = CIP (constant intensity and constant photoperiod), T2 = CIDP (constant intensity with dynamic photoperiod), T3 = DICP (dynamic intensity with constant photoperiod), and T4 = DIP (dynamic intensity and photoperiod). Rows represent measurements collected during Week 1, Week 2, Week 3, and the overall average across weeks. T1 and T2 maintained constant PFD throughout the experiment, whereas T3 and T4 applied gradually increasing PFD across the three-week treatment period. T2 and T4 additionally included gradually increasing photoperiods during the same period. Values within cells represent measured PFD (µmol m⁻² s⁻¹), and color bars indicate the corresponding PFD intensity range.
